# Integrating pediatric TB services into child healthcare services in Africa: study protocol for the INPUT cluster-randomized stepped wedge trial

**DOI:** 10.1186/s12889-020-08741-2

**Published:** 2020-05-06

**Authors:** Lise Denoeud-Ndam, Rose Otieno-Masaba, Boris Tchounga, Rhoderick Machekano, Leonie Simo, Joseph Phelix Mboya, Judith Kose, Patrice Tchendjou, Anne-Cécile Zoung-Kanyi Bissek, Gordon Odhiambo Okomo, Martina Casenghi, Jennifer Cohn, Appolinaire Tiam, the INPUT Study Group, Millicent A. Ouma, Armel Zemsi, Saint Just Petnga, Stephen Siamba, Eric Youm, Leila Katirayi, Justine J. Odionyi, Peter J. Dodd, Nyashadzaishe Mafirakureva, Oluwarantimi Adetunji, Sushant Mukherjee

**Affiliations:** 1 EGPAF, Geneva, Switzerland; 2 EGPAF, Nairobi, Kenya; 3 EGPAF, Yaounde, Cameroon; 4 EGPAF, Washington DC, USA; 5 Erasmus University Medical Centre, Rotterdam, the Netherlands; 6 Ministry of Public Health, Yaounde, Cameroon; 7 Ministry of Health, Homa Bay, Kenya; 8 University of Bergen, Bergen, Norway

**Keywords:** Tuberculosis, Child, Case detection, Effectiveness, Cluster-randomized trial, Implementation, Stepped-wedge

## Abstract

**Background:**

Tuberculosis is among the top-10 causes of mortality in children with more than 1 million children suffering from TB disease annually worldwide. The main challenge in young children is the difficulty in establishing an accurate diagnosis of active TB.

The INPUT study is a stepped-wedge cluster-randomized intervention study aiming to assess the effectiveness of integrating TB services into child healthcare services on TB diagnosis capacities in children under 5 years of age.

**Methods:**

Two strategies will be compared: i) The standard of care, offering pediatric TB services based on national standard of care; ii) The intervention, with pediatric TB services integrated into child healthcare services: it consists of a package of training, supportive supervision, job aids, and logistical support to the integration of TB screening and diagnosis activities into pediatric services. The design is a cluster-randomized stepped-wedge of 12 study clusters in Cameroon and Kenya. The sites start enrolling participants under standard-of-care and will transition to the intervention at randomly assigned time points. We enroll children aged less than 5 years with a presumptive diagnosis of TB after obtaining caregiver written informed consent. The participants are followed through TB diagnosis and treatment, with clinical information prospectively abstracted from their medical records.

The primary outcome is the proportion of TB cases diagnosed among children < 5 years old attending the child healthcare services. Secondary outcomes include: number of children screened for presumptive active TB; diagnosed; initiated on TB treatment; and completing treatment. We will also assess the cost-effectiveness of the intervention, its acceptability among health care providers and users, and fidelity of implementation.

**Discussion:**

Study enrolments started in May 2019, enrolments will be completed in October 2020 and follow up will be completed by June 2021. The study findings will be disseminated to national, regional and international audiences and will inform innovative approaches to integration of TB screening, diagnosis, and treatment initiation into child health care services.

**Trial resistration:**

NCT03862261, initial release 12 February 2019.

## Background

### Childhood tuberculosis

In 2016, more than 1 million children (< 15 years) had tuberculosis (TB) disease worldwide over 253,000 of whom died, making TB one of the leading causes of death in children [1, 2]. Younger children and children with HIV co-infection are at the highest risk of progressing to disease after infection, developing disseminated forms of TB and dying [3–5]. Africa accounts for about one-third of all pediatric TB cases with an incidence of 29–34/100,000, which is double the global average [2, 6].

The main challenge in childhood TB is the difficulty in establishing an accurate diagnosis of active TB since symptoms are not specific, children cannot easily produce sputum, and they mostly develop paucibacillary disease [5, 7]. The Xpert MTB/RIF assay is a major advance for diagnosis of TB [8]. Sensitivity of Xpert is lower in children than in adults, however the assay does help to increase the proportion of children with a laboratory-confirmed diagnosis of TB [9]. However, its use is limited by children’s difficulty to produce sputum. These challenges in case detection and diagnosis are the main reasons why only 39% of estimated pediatric TB cases are notified to national TB programs (NTPs), with the remaining children undiagnosed or unreported [2].

### Rationale for the INPUT study

In many sub-Saharan African countries, diagnosis and treatment of children with active TB is centralized at the high level of care and provided in separate TB units. This adds to the list of barriers to identification of cases and scale up of treatment of children with TB disease. Pediatric services such as Maternal, Neonatal and Child Healthcare (MNCH)/under-5 services, outpatient clinics for children, pediatric ART services, and nutrition rehabilitation services may represent a first entry point for children with TB disease in many countries. The importance of linking TB prevention and care to maternal and child health programs has been recognized [10].

Yet, very limited data are available on the feasibility of integration of TB care in the child health care services and its impact on pediatric TB case detection, cascade of TB care, and treatment outcomes. Data from the HIV context have demonstrated that integration of HIV services into other health services is feasible, does not compromise care and results in lower rates of loss-to-follow-up [11, 12]. Similarly, there is growing evidence that integrating TB services into HIV services results in good quality care and improved retention in adults [13, 14]. In a recent Cochrane review synthesizing available evidence on the impact of interventions to increase tuberculosis case detection in adults at primary healthcare or community-level services, the authors reported an increase in TB case detection in short term [15]. The effect of active case finding on treatment outcome needed further evaluation since most studies included in Cochrane were not powered to answer this question.

Further, the DETECT study in Uganda evaluated a mixed intervention combining decentralization of TB care in primary health care facilities and implementation of TB screening at community level. It showed that the strengthening of TB services at peripheral health facilities was associated with increased case finding, improved treatment outcomes and the successful implementation of contact screening and management [16].

However, evidence on the integration of TB care in other child health services is limited. One retrospective study showed the feasibility of intensified case finding efforts in children at nutrition rehabilitation centers. They found that out of the 440 children with severe acute malnutrition screened, 39 (8.8%) were diagnosed with TB. Among these, 87% initiated TB treatment [17]. A study in Ethiopia examined intensive screening of children under-5 years old in MNCH clinics and the results demonstrated the feasibility of this approach, though its impact could not be assessed due to the absence of a comparison group [18]. A recent publication assessed the impact of systematic verbal screening at pediatric outpatient department, with clinical evaluation and free diagnostics, on childhood TB detection in rural Pakistan. These activities resulted in a three-fold increase in pediatric TB case detection, thus showing that this strategy can identify children with TB who may otherwise be missed in rural health settings [19].

## Methods/design

### Hypothesis and aims

We hypothesize that the integration of TB services into non-TB pediatric healthcare services; through increasing the capacity of all health care workers to diagnose children with TB, improving intra and inter-facility linkages and coordination and improving patient-centered care and support, will result in improved case detection and treatment outcomes in children less than 5 years of age. We define TB services as active case finding, diagnosis of active TB disease and initiation on treatment fr drug-sensitive TB disease.

The primary objective of this study is to assess the effect of integrating TB screening, diagnosis and treatment services in child health care services compared to standard of care in the second level hospitals (hubs) and their attached health centers (spokes), on the proportion of TB cases diagnosed among children < 5 years old (the number of children who are clinically or bacteriologically diagnosed with TB over the total number of children attending the child healthcare services).

Secondary objectives are:

- To compare the outcomes of the intervention (integrating TB services intervention into child health care services) compared to routine, standard of care on the cascade of care for diagnosis of drug sensitive tuberculosis (DS-TB) and treatment initiation and completion, overall and disaggregated by pediatric health services and level of care
- To determine the number of children needed to screen in order to diagnose one pediatric TB case through integration of TB services in various child health services.
- To describe the characteristics of children diagnosed with Active TB disease.
- To describe WHO-defined TB treatment outcomes and associated factors.
- To determine the rate of HIV infection among children with presumptive and confirmed pediatric TB diagnosis.

Qualitative objectives are:

- To assess caregivers’ acceptability and perceptions about integrated pediatric TB services;
- To understand the effect of the integrated TB service model on service delivery in the non-TB pediatric health services;
- Through community workers, to explore the community knowledge about pediatric TB and perceptions about the integrated pediatric TB services.

A cost-effectiveness analysis will estimate the costs and benefits associated with TB care under the intervention and standard of care and determine incremental cost effectiveness of the intervention, both from a health system and patient perspective.

### Study design

We are conducting a multi-country stepped-wedge cluster-randomized controlled trial to compare two strategies for case detection and management of tuberculosis in children under the age of 5 years: i) The standard of care offers pediatric TB services based on current routine approach (national standard of care); ii) The CaP TB intervention will offer pediatric TB services integrated into key child healthcare services.

In this study, a stepped wedge design [20] has been preferred to parallel cluster trial for the following reasons: It allows every site participating in the study to eventually be exposed to the CaP TB intervention, which is in line with the scale up of CaP TB activities, and more acceptable for the MoHs. The phased roll-out of CaP TB activities provides logistical advantages. The intra-cluster correlation is anticipated to be high and cluster size large so that a stepped wedge design is likely to be more efficient than the simple parallel cluster design [21], and will require a smaller number of clusters [22]. Finally, stepped-wedge design allows for the control of trends over time due to external changes in the way care is delivered, for example changes in national guidelines.

In INPUT study, the randomization unit or cluster is defined as a district or sub-district hospital (the hub) and some of its attached primary health care centers (spokes) that refer patients or samples to these facilities for TB diagnosis. A randomization list prepared by the biostatistician in EGPAF determines time of enrollment of each of these sites into the intervention phase.

**Fig. 1:**
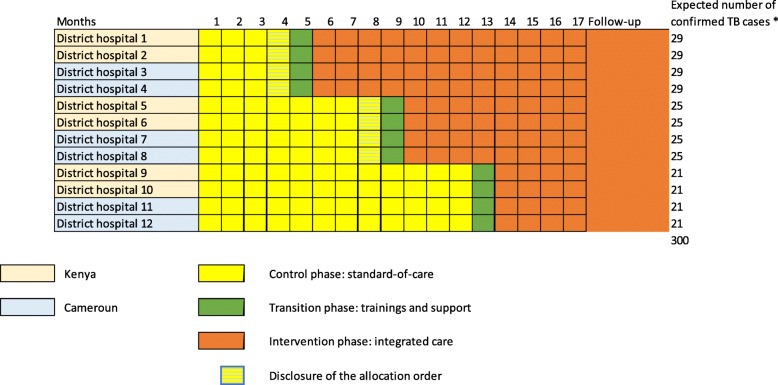
INPUT study stepped-wedge design. *Estimation based on the hypothesis of 2 cases diagnosed per 1000 children seen under standard-of-care and doubling under intervention

Figure 1 shows the stepped wedge study design. Justification for total number of steps, number of clusters per step and duration of steps is given in the “sample size” section.

### Study sites and participants

The study is being implemented in Kenya and Cameroon. These countries were purposively selected based on the TB burden, geographical location and representativeness of the epidemic in sub-Saharan Africa.

The planned total number of sites is 12 hospitals (hubs), 6 in each country, with up to two purposively selected health centers (spokes) per hospital selected based on their TB case load and accessibility from the hub.

Study hubs were purposively selected in collaboration with the NTPs and MoH in Cameroon and Kenya according to the following criteria:

- Sites where regions or counties where CaP TB was being implemented.
- At least 6000 pediatric visits per year altogether in the district/county hospital and related selected health centers. Based on available data in the two countries, we assumed each site may be able to see on average 20 children with presumptive TB per month and diagnose at least 12 pediatric TB cases per year (one per month), under the standard-of-care.
- Routine adult TB diagnosis and treatment available onsite.
- No previous or ongoing introduction of interventions for integration or increasing coverage of TB services, apart from NTP interventions that are considered as standard-of-care throughout the whole study duration.

According to these criteria, the selected district hospitals in Cameroon are Soa, Akonolinga, Loum; Mbouda, Dschang and Foumban. In Kenya, the selected district or sub-district hospitals are Ndhiwa, Kendu, Homa Bay county hospital, Kakuma mission hospital, Lopiding subcounty hospital and Lokitaung’s subcounty hospital. Each study site is allocated a unique site identification number.

The study population consists of all the children aged less than 5 years who present for care in the selected study district hospitals and selected attached heath centers. These children serve as the denominator to calculate the proportion of cases diagnosed in child healthcare services in each facility (the primary outcome of the study).

Children who meet all eligibility criteria are eligible for enrolment in the prospective follow-up of the study after the study nurse obtains written informed consent from caregivers.

Inclusion criteria are:

- Children < 5 years old.
- Presumptive pulmonary or extra-pulmonary TB case: symptoms or clinical signs suggestive of active TB disease.
- TB diagnostic investigations initiated.
- Commitment to take treatment in the clinic of enrolment or another INPUT study site.
- Parental/caregiver consent for the child to participate in the study.

Exclusion criterion is:

- Children who are TB contacts but without symptoms or signs of active TB.

### Standard-of-care of TB services in study sites

In both countries, TB care is currently delivered in one place called the TB unit or TB clinic. In Cameroon, TB services are offered only at health facilities where a doctor is in service and TB screening is not currently recommended nor done systematically at each entry point. While in Kenya TB services are offered at primary health care level, TB screening is recommended at every entry point, children and adults with presumptive diagnosis of TB all go to TB unit for diagnostic work-up and treatment.

According to national guidelines in Cameroon and Kenya, TB diagnosis in children involves the screening of children who present in health facility for symptoms of active TB disease: non-remitting cough for more than 2 weeks, fever for more than 10 days, night sweats for more than 2 weeks, fatigue, reduced playfulness or decreased activity, weight or appetite loss or failure to thrive during the last 3 months. Symptomatic children are identified as TB presumptive and should start TB investigations in the TB clinic, including routine specimen collection and testing. In Kenya, Xpert testing is available as standard-of-care in most district hospitals, this is not the case in Cameroon.

Children with presumptive TB and for whom all TB laboratory diagnostic tests were negative or investigations were not done, undergo clinical and if possible radiological investigation as per national guidelines in Cameroon and Kenya. Ultimately, the clinician in the TB clinic can make a clinical diagnosis of TB based only on history and physical examination findings, including clinical response to a trial of broad-spectrum antibiotics. Treatment initiation and follow-up take place in the TB clinic.

### Intervention under evaluation: the CaP TB intervention package

The CaP TB intervention has been defined for every level of care. It involves a package of training, supportive supervision, monitoring tools, job aids and logistical support (e.g. creation or strengthening of referral networks, sample transport) for the integration of TB activities into non-TB child healthcare services.

These components are specifically oriented towards integration of screening and diagnosis services into child health care services:

- Integration of the screening into all the child health care services with introduction of a specific case detection tool and updated presumptive TB register.
  - To improve symptom screening in the facilities, trained cough monitors are placed in all the study sites and serve as lay health workers who screen patients in waiting rooms. They accompany children with presumptive TB diagnosis to health care providers.
- Improvement of diagnosis capacity (according to the national guidelines or WHO/UNION recommended approach to TB diagnostic in children [23, 24] and their integration in all levels of care and all services:
  - Strengthened use (in Kenya where it already exists) or introduction (in Cameroon where it needs to be developed) of a diagnostic algorithm for the diagnosis of TB in children, especially at primary health care level, where capacities for biological diagnosis are often limited.
  - Trainings for specimen collection, including gastric aspirates, nasopharyngeal aspirates, induced sputum and lymph node aspiration, especially at district hospital level, with integration into the different pediatric entry points including the inpatient ward for hospitalized children.
  - Support for Xpert testing of all the specimen collected, either on site or through sample referral, with procurement of GeneXpert devices and Xpert cartridges.
  - Support for X-rays completion: use of transport vouchers for children to go to the referral site for X-ray if needed.

Children who are diagnosed with TB disease are initiated on anti-TB treatment at the point of diagnosis. Drug dispensation, drug refill and follow-up take place at the TB unit in hub sites, or as designed through routine system in spoke sites.

### Sample collection methods and concomitant care

Sputum and other laboratory specimens such as gastric lavage, induced sputum, nasopharyngeal aspirate and lymph node aspiration are collected from children and processed according to manufacturer protocols and national guidelines.

Specimens are tested using Xpert MTB/RIF assay (Cepheid, Sunnyvale, California, USA). Xpert testing and interpretation of results are carried out according to manufacturer instructions. Sputum smear microscopy is performed following the Ziehl-Nielsen method. At least 100 high power fields will be read before returning a negative result. Results are reported as follows: No acid-fast bacilli seen, exact number of bacilli seen if less than 10 in 100 fields, + if 10–100, ++ if 100–1000, +++ if > 1000 per 100 fields.

Sputum samples for culture are processed according to national standard of care. This is not part of CaP TB project but will be carried out with support from national program.

All children who have positive TB symptoms and who are HIV exposed or whose exposure status is unknown will be screened for HIV with age-specific test. For children who are still breastfeeding or young infants aged less than 12 months, HIV diagnosis will be done using the Cepheid/Alere point of care qualitative test while older children who have stopped breastfeeding for more than 6 weeks will be tested for HIV using rapid test according to national standard of care. All HIV laboratory testing will be performed per the routine MoH protocols.

Treatment of active TB will follow national and WHO guidelines during both the standard-of-care and intervention phase. During intensive phase of treatment and as per the current WHO guidelines the treatment will be dispersible fixed dose combinations of isoniazid-rifampicin-pyrazinamide associated with ethambutol and during the continuation phase the treatment will be isoniazid-rifampicin. The duration of the treatment will depend on the type of TB disease (pulmonary or extra-pulmonary) and will follow national guidelines. Typically, the treatment will last 6 months in pulmonary TB and will extend to 1 year for TB meningitis or osteo-articular meningitis.

Although not part of the study, MDR and/or XDR TB cases and cases needing additional care will be treated in accordance to national guidelines. Whenever possible, patients with confirmed MDR and/or XDR TB diagnosis will be documented in order for the study team to calculate the rate MDR/XDR among the children.

### Evaluation activities and data collection processes

Trained study nurses are present in each study cluster. They are responsible for the screening, consent and enrolment processes, collection and maintenance of study records and data, in the hubs and their related spokes.

**Fig. 2:**
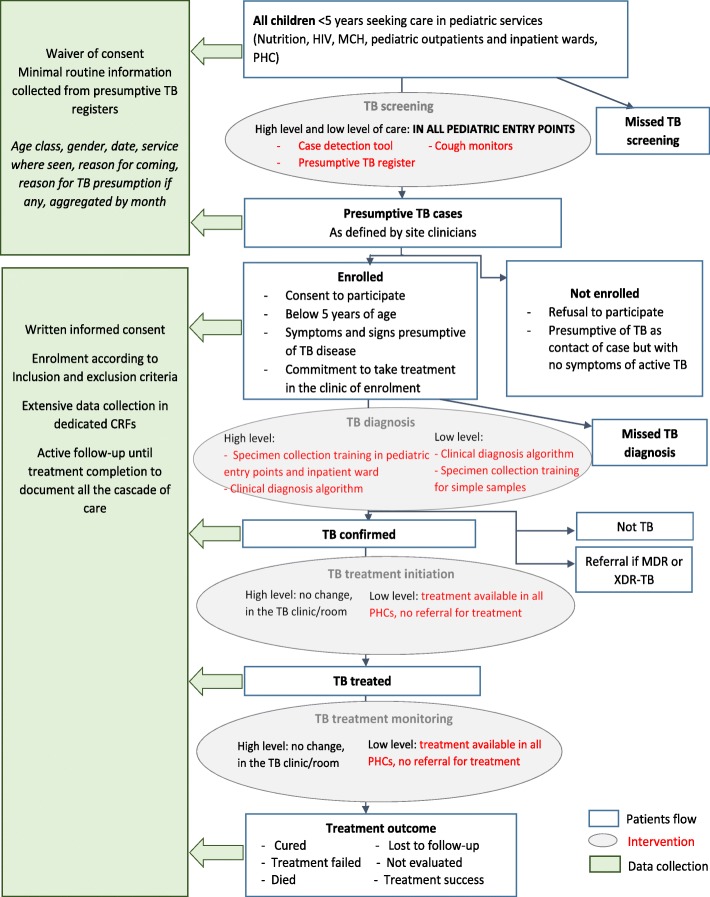
INPUT study flow-chart

Different processes for data collection have been identified, as shown in the study flow-chart (Fig. 2). For children attending pediatric health care services, routine aggregated data are collected to define the number of children attending the facility. This will serve as denominator to calculate the proportion of cases diagnosed in the facility.

**Fig. 3:**
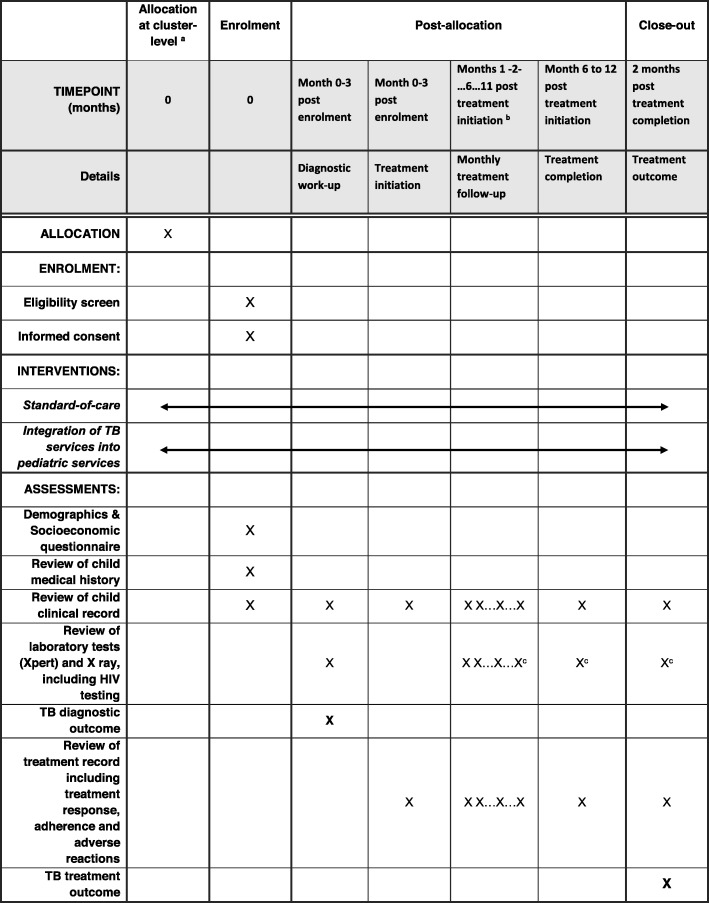
INPUT study SPIRIT flow diagram. **a** Allocation done at cluster level prior to enrolment of  participants, according to stepped-wedge design. **b** Ideally, simple TB infection treatment will last for six months, but for TB meningitis, osteo-articular TB or cases that do not respond to treatment appropriately, treatment may be extended to 12 months. All Information on treatment follow-up visits will be recorded as they occur. At least monthly visits will be planned. Information on additional visits, when the child comes for intercurrent illnesses, will also be recorded. **c** If any new results are collected

The second process involves prospective, longitudinal follow-up for children with presumptive diagnosis of TB, until confirmed diagnosis, treatment initiation and completion. Figure 3 (SPIRIT diagram) outlines the study procedures for these children. This prospective follow-up involves a specific electronic case report form to document all the cascade of TB care. Data clear from identifiable information are collected and entered using electronic tablets by direct data entry into a database that has been designed specifically for this study and stored on a secure web-based server.

In both standard-of-care and intervention phases, site clinicians notify the study nurse of all children potentially eligible for enrolment. In addition, the study nurse regularly reviews the existing registers in the sites to extract anonymous screening information about children with presumptive TB: service where seen, age, gender, reason for coming, date of visit. Study nurses approach mothers or caregivers of children who have a diagnosis of presumptive TB on the day of presentation or as soon as possible thereafter to screen the child for eligibility as per inclusion criteria and enroll them after obtaining written consent.

Then, diagnosis, treatment and monitoring activities are scheduled according to the routine of TB care. The study nurse collects data prospectively upon diagnostic work-up (clinical, laboratory and X-ray findings if done). Children are initiated on treatment by the healthcare providers on site and the study nurse prospectively collects data from treatment reports. Similarly, children coming monthly to the study sites for treatment refill are monitored by the healthcare providers as usual, and the study nurse takes this opportunity to collect follow-up data. Ideally, follow-up will continue until 2 months after treatment completion but the maximum follow-up duration is 12–14 months. The duration of follow-up is determined at treatment initiation based on the type of TB diagnosis. However, some reasons may lead to the interruption of monitoring (referral of the child, serious adverse event, death, loss to follow-up). In all cases, the date and reasons for the interruption will be collected in the report form.

Enrolled children who are determined not to have TB are followed during diagnostic procedures then terminated from the study once they are determined not to have active TB.

For children with a missed appointment for TB routine services, the in-country routine procedures for tracing patients on TB care are followed, including phone contact, household visit of community workers, or any other mean according to national guidelines. In addition to these routine procedures implemented by the health facility staff, the study nurse also keeps a contact log with phone numbers (consent is sought for this) and may contact the caregiver to remind him of the appointment in TB care. If the child does not attend the routine TB visit within a month of the planned schedule, the visit is recorded as a missed visit.

In case the child comes to the study site for an unscheduled visit caused by an illness during his follow-up, the study nurse collects clinical and laboratory data from medical reports to document the nature and severity of the disease.

The study nurses also collect information on how the TB activities are being managed by the healthcare providers on site, both during the standard-of-care and the intervention period, as a measurement of the fidelity of implementation. A specific log had been designed for this purpose where deviations between practice and guidelines are recorded and shared prospectively with the CaP TB program team in order to improve practices.

### Outcomes

The primary outcome is the proportion of children diagnosed with TB defined as the number of pediatric TB cases diagnosed over the number of children attending the child healthcare services during the study period.

Main secondary outcomes include:

- Proportion of children screened for TB among the total number of children attending the child healthcare services.
- Proportion of children diagnosed with TB (bacteriologically or clinically) among presumptive TB cases, overall and disaggregated by HIV status and nutrition status.
- Time from screening to clinical or bacteriologic diagnosis.
- Time from diagnosis to treatment initiation.
- Proportion of cases with a bacteriologically confirmed diagnosis.
- Treatment outcomes for patients initiated on treatment according to WHO categories, with a focus on treatment completion rate.
- Cost-effectiveness from a health system perspective, as US dollars per disability-adjusted life-year (DALY) averted.
- Comprehensive understanding of caregivers’ perceptions and patients’ and communities’ acceptability of receiving integrated pediatric TB services within pediatric care.

### Adverse events

Parents/caregivers are informed of any side effect which could be associated with TB diagnosis procedures, especially invasive sample collections (gastric aspirate, lymph node fine needle aspiration, CSF collection, ascites collection, nasopharyngeal aspirate), and with TB treatment.

The investigators describe adverse events occurring during follow-up of children in a specific section of the case report form.

Serious adverse events as defined by ICH [25] or unexpected adverse events are reported to the ethics committees as per their guidance.

Since drugs dispensed during the study are part of national standard of care, children who develop adverse reactions are managed according to national standard of care, according to the national pediatric TB guidelines in Kenya and ad-hoc guidelines in Cameroon. The study nurse collects outcome data from clinical and laboratory reports.

### Risks and benefits for participants

Patients who utilize CaP TB supported facilities in the intervention phase benefit from better access to TB services as part of the general CaP TB program implementation. There is no additional benefit to study participants.

Risks to all participants is minimal. Potential risks include the potential for unintended disclosure of mother/caregiver and infant’s TB infection status and for those who are HIV infected their HIV status.

Children enrolled in the INPUT study are identified by their unique study identifier, and data collection and forms are linked to the patient using this unique identifier only. All study documents are maintained by the study team in a locked cabinet in an office with limited access. Following study completion and all analyses, paper-based study documents for all data collection activities will be destroyed after 7 years.

Caregivers may also feel discomfort in answering some of the interview questions such as those to child’s exposure to TB infection at home. Interviews are conducted in private areas free from general view and out of hearing reach. Study staff is thoroughly trained on interviewing techniques in general and those related to this study in particular.

### Sample size

The total number of clusters enrolled in this study has been determined under three assumptions: 1) the expected “sample size” of clusters, which is the number of children seen per month in each cluster; 2) the expected baseline “detection rate”, which is the proportion of cases diagnosed on the total number of children, during the standard-of-care phase; and 3) the “effect size” of the intervention which is the expected increase in detection rate between standard-of-care and intervention phase. According to baseline records, we estimate that the number of consultations in children less than 5 years of age will be at least 500 per month in each cluster. We assume that at least 2 children will be diagnosed with TB per 1000 children attending the child health care services, under standard-of-care. Published studies on interventions to enhance case detection in adults demonstrated an effect of the intervention of 50% [15]. However, it is well known that under-reporting of child TB cases is much more prevalent than adult TB cases [2], and thus we believe it will be more subject to improvement. Indeed, whereas the incidence of TB in children is expected to be 10 to 12% of the total number of TB cases in a particular country, the notified TB cases in children represents only 6% of the total number of TB cases in Cameroon and Kenya. We designed the study according to an expected effect size ranging between 50 and 100%.

Based on a stepped-wedge design, with 2000 children attending consultations during each four-month step of the study, four randomly selected study sites per step and 12 sites in total (three steps in the study), and an ICC correlation of 0.001, we will have 96.5% power to see a doubling in the case detection from two to four per 1000.

With this design and assumptions, we expect to follow 300 confirmed pediatric TB cases during the whole study duration. Current TB treatment completion rates in routine care are estimated at least at 70%. With 96 cases expected under standard-of-care and 192 cases under intervention, the power will be 80% to detect a 20% increase in completion rate.

### Randomization and blinding

The randomization sequence was generated by the study biostatistician using Stata 15.0 statistical software, with stratification by country. In each country, two sites were allocated to cross into the intervention after 4 months under standard-of-care phase, 2 after 8 months under standard-of-care, and 2 after 12 months under standard-of-care. The allocation sequence remains concealed from all investigators during the first 3 months of the study, so that all sites start under standard-of-care blinded of when the intervention phase will start. Then, the biostatistician discloses to Principal Investigators which four sites in the two countries have been randomized to start the intervention in the first step (see Fig. 2). The four sites undergo a transition phase of 1 month with trainings before crossing to the intervention phase. All other sites continue in the standard-of-care, still blinded on the time when they will start the intervention, until the last month before the next step. The one-month notice given before starting into the transition phase will allow sites to adequately plan for trainings in the study sites.

The study design does not allow the site investigators to be blinded after assignment to interventions.

### Statistical considerations

In a stepped wedge study, exposed (intervention) and unexposed (standard-of-care) observation periods take the place of “arms” in parallel cluster trials. Thus, the distribution of outcomes across unexposed observation periods is compared with that across the exposed observation periods [20, 21].

Characteristics of the individuals and clusters will be summarized by exposure status (intervention versus standard-of-care period) to assess for potential selection biases and lack of balance. These characteristics will also be compared by steps. This will include the numbers analyzed, the average cluster size, cluster characteristics (in terms of TB care cascade), and important patient characteristics (TB treatment outcomes). All comparisons will be performed with a level of significance alpha of 5%.

Following an intention to treat principle, clusters will be analyzed according to their randomized crossover time irrespective of whether crossover was achieved at the desired time. In addition, we will carry on per protocol analyzes where the clusters will be analyzed according to the real timing on which the crossover actually occurred.

Data collected during the one-month transition phase from standard-of-care to intervention will not be considered in the analysis of outcomes, in order to minimize contamination between study periods.

We will estimate the TB detection rates for each cluster during each step. We will then summarize the detection rates by intervention period (standard-of-care versus integration) and display the summaries using graphs. To estimate the effect of integration on TB detection rates (i.e., proportion of cases diagnosed), we will use generalized linear mixed Poisson models. The effect will be presented as rate ratio and associated 95% confidence interval.

We will use time to event survival analysis approaches with frailties to estimate the effect of the intervention on treatment completion. This approach allows us to account for TB patients lost to follow-up or who transfer to other facilities while estimating treatment completion rates. In addition, we will use descriptive analysis to assess the fidelity of the implementation of the intervention over time and secondary outcomes that are likely to be infrequent and thus with limited power to detect differences.

### Cost-effectiveness and qualitative components

Methods used for the qualitative component and the cost-effectiveness component of the study are not captured in this manuscript. The latter are detailed in a separate Health Economics Analysis Plan [26].

### Time period for the study

Participants are planned to be recruited from May 2019 until October2020, and follow-up will be completed by June 2021. Then, results will be analyzed and shared as described in dedicated parts of the protocol, with publications expected end 2021 / early 2022. The study is planned to last for 3 years overall.

## Discussion

The INPUT stepped-wedge cluster-randomized trial has the potential to provide new and strong evidence on the effectiveness and feasibility of innovative approaches to integration of child TB care into pediatric entry points.

To ensure the robustness of the study, good clinical practice requirements will be met. Research staff in the field is under the supervision of the country principal investigator, who provides direct oversight and monitoring of specific research activities. Before the study starts, the study research teams has been trained on the protocol, SOPs, data collection and standard study monitoring tools. All study team members were trained in Human Subjects Protection and signed a confidentiality agreement prohibiting disclosure to non-study team members of any individual-level information including TB diagnosis and HIV status.

The study has a detailed monitoring plan involving regular staff visits to project sites and quality assurance activities related to the routine project activities. The global PI, global co-investigator/trial manager and the regulatory officer carry out regular study monitoring to ensure the study is being implemented with fidelity to protocol.

A Scientific Advisory Committee (SAC), made of pediatric TB experts who are external and independent from all project collaborators, provides scientific oversight to the study and reviews the study progress regularly.

We have identified a few risks and limitations pertaining to the study implementation.

MoHs in the two countries may be willing to start interventions to promote active case finding (as already in the implementation phase in Kenya) and integrated TB services, as recommended by WHO. These national activities will not be slowed down in the study sites. Information regarding the standard-of-care and its evolution throughout the standard-of-care period will be carefully reported. The stepped-wedge design will allow taking into account temporal trends which could result in these standard-of-care changes during the pre-implementation period.

Chart abstraction data depends on the quality of data collected by facilities as part of routine data collection. Thus, it is possible we will have missing or incomplete data for some of the records in the pre-intervention phase. Our staff is available to support facility based health workers in collecting data as requested and we are monitoring carefully the quality early on in the process to correct any quality issues that may arise.

We calculated the sample size according to an optimistic estimate of the number of pediatric TB cases diagnosed, either in the standard-of-care or intervention phase. If the number of cases is lower than expected, the study may be underpowered to show the impact of the intervention on TB case detection.

Finally, an initial increase in case finding may be observed at study initiation because we may capture prevalent cases not diagnosed since then, though this effect may be less pronounced than in adult studies. We also expect that the start of the study, with the use of new TB registers and M&E forms, will be associated with increased awareness about TB and may thus improve TB diagnostic access, even before roll-out of the intervention. This confounding effect is likely to be unavoidable and will need to be taken in consideration while interpreting results.

In summary, the INPUT study uses a robust methodology and its results are expected to inform innovative strategies on organization of TB care. The study results will be shared with national and international clinicians, program implementers, scientific audiences and policy makers through reports, presentations and manuscripts. Demonstrating a benefit of integrated child TB care on TB case detection will provide strong supportive evidence to catalyze the widespread implementation of this intervention across Africa.

## Data Availability

Not applicable.

## Abbreviations

ART: Antiretroviral therapy
CNERSH: Cameroon national ethics committee for research in human health
CSF: Cerebrospinal fluid
DS-TB: Drug sensitive tuberculosis
EGPAF: Elizabeth Glaser Pediatric AIDS Foundation
ERC: Ethics Review Committee
HIV: Human Immunodeficiency virus
ICH: International Council for Harmonization
INPUT: Integrating Pediatric TB services into child healthcare services in Africa
IRB: Institutional Review Board
KNH UON: Kenyatta National Hospital-University of Nairobi
M&E: Monitoring and Evaluation
MCH, MNCH: Maternal (Neonatal) and Child Healthcare
MDR-TB: Multidrug resistant tuberculosis
MoH: Ministry of Health
NTP: National Tuberculosis Program
SAE: Serious Adverse Event
SOP: Standard Operating Procedure
TB: Tuberculosis
The UNION: International union against tuberculosis and lung disease
US, USA: United States of America
WHO: World Health Organization
XDR-TB: Ultra drug resistant tuberculosis
Xpert, Xpert MTB/RIF: assay for detecting *Mycobacterium tuberculosis* (MTB)and rifampicin (RIF) resistance using a rapid PCR-based diagnostic platform (Gene-, Cepheid, Sunnyvale, California, USA)
